# Supplementary material for: Microtubule-independent movement of the fission yeast nucleus
Source: J Cell Sci. 2021 Mar 26;134(6):jcs253021. doi: 10.1242/jcs.253021 (PMC8015250; doi:10.1242/jcs.253021)
Supplement: Supplementary information [file joces-134-253021-s1.pdf]

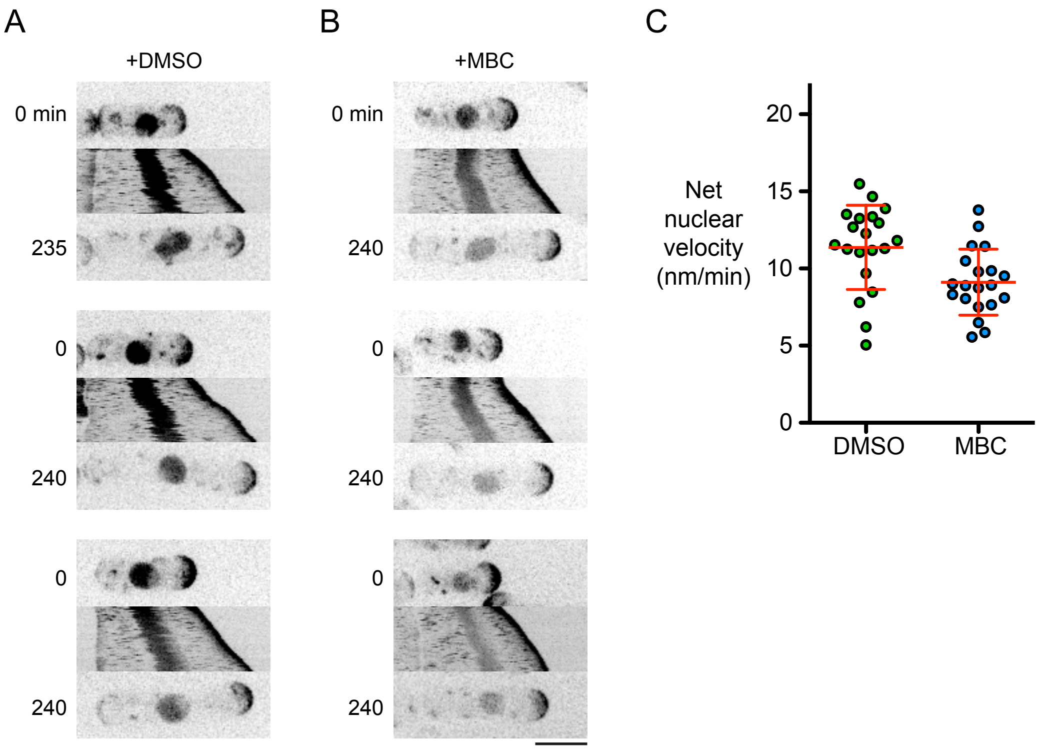

Figure S1. MINM occurs in cells expressing reporters other than CRIB-3mCitrine. **(A, B)** Timepoints and kymographs of nuclear movement from movies of DMSO-treated **(A)** and MBC-treated **(B)** cells expressing GST-NLS-mCherry as a nuclear marker and mCherry-Bgs4 as a cell tip-growth marker. Timepoints correspond to beginning and end of kymographs (min). Three examples are shown for each condition. In the top example in A, the 235 min timepoint is shown because the cell initiated mitosis at 240 min. **(C)** Net nuclear velocity towards growing cell tips in monopolar-growing DMSO- and MBC-treated cells. Red lines show mean and SD (n=20 for each condition). Net nuclear velocity in MBC is slightly lower than in DMSO (*t*-test, *p*=0.006); in cells expressing the CRIB-3mCitrine reporter, the difference in net nuclear velocity in MBC vs. DMSO was not statistically significant (Fig. 1G). Bar, 5 μm.

Journal of Cell Science • Supplementary information

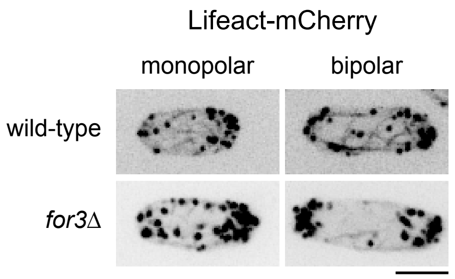

Figure S2. Actin organization in *for3*Δ mutants.
Lifeact-mCherry images of interphase monopolar- and bipolar-growing wild-type and *for3*Δ cells. Note that cortical actin patches are much more intense than actin cables. Bar, 5 μm.

Journal of Cell Science • Supplementary information

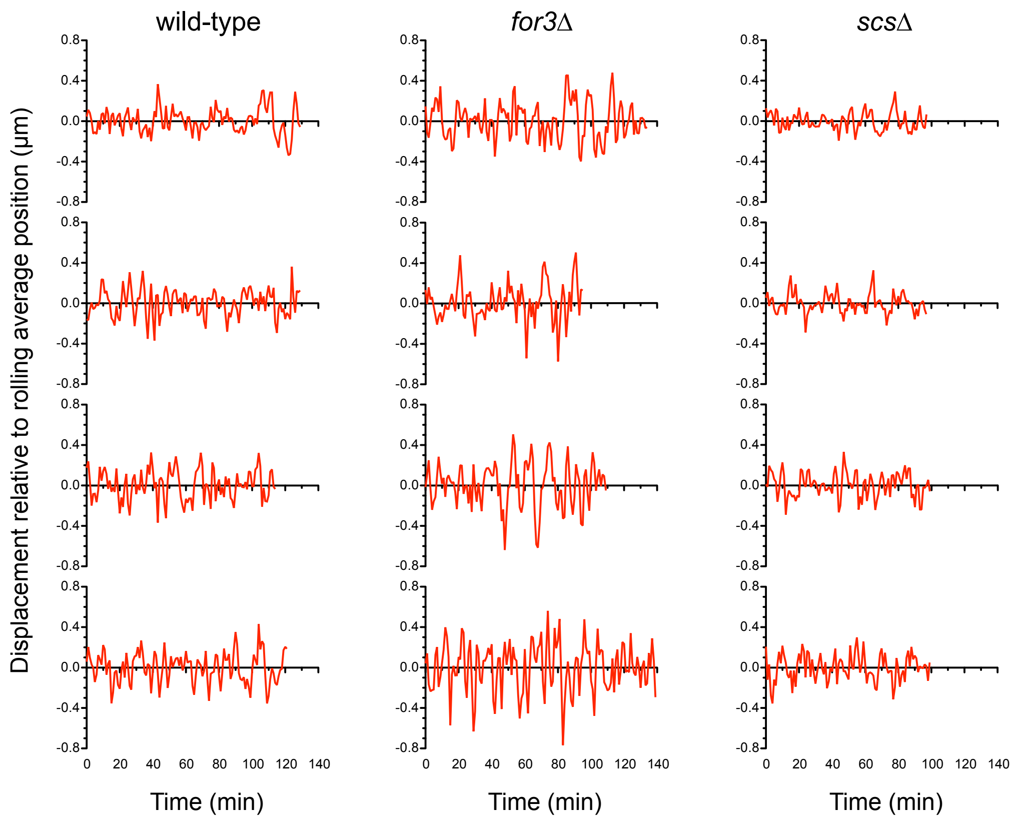

Figure S3. Fluctuations in nuclear position in untreated wild-type, *for3*Δ and *scs*Δ cells. Traces show examples of displacement of the nucleus relative to its rolling average position during normal growth, after detrending (see Materials and Methods). Individual traces correspond to individual root-mean-square (RMS) displacement datapoints in Fig. 6D. Within each genotype, traces are shown from top to bottom in ascending order of RMS displacement; RMS displacements for the examples shown are: 0.125, 0.133, 0.150, and 0.152 μm (wild-type); 0.184, 0.187, 0.221, and 0.240 μm (*for3*Δ); and 0.089, 0.097, 0.123, and 0.132 μm (*scs*Δ). Times of traces range from 96 to 140 min.

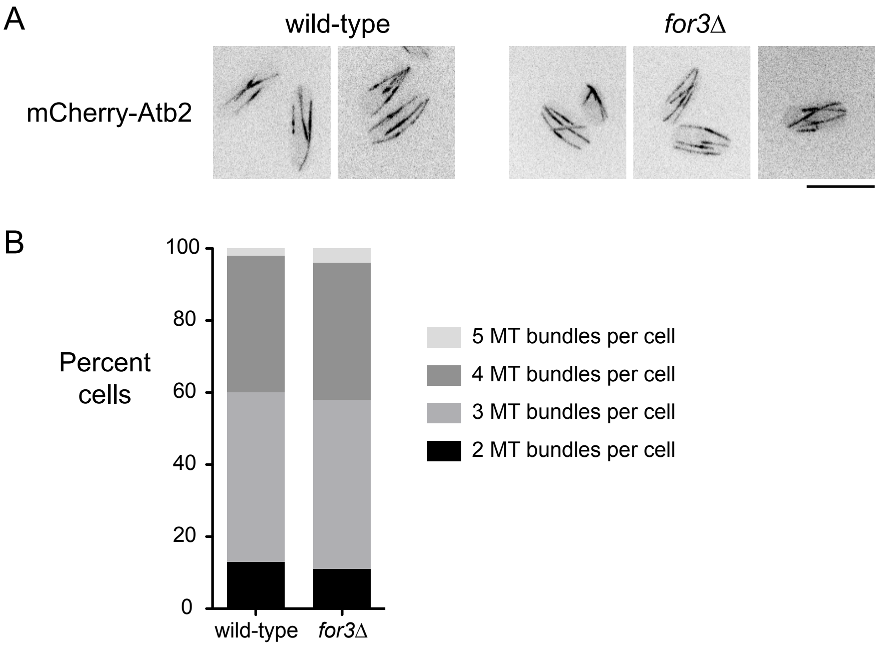

Figure S4. Interphase microtubules in wild-type and *for3*Δ cells.
(A) mCherry-Atb2 (alpha-tubulin)-labeled microtubules (MTs) in wild-type and *for3*Δ cells. **(B)** Quantification of numbers of microtubule bundles per cell, from images as in A. n=189 wild-type cells and 56 *for3*Δ cells measured. Differences were not statistically significant (chi-square test, p=0.9). Bar, 10 μm.

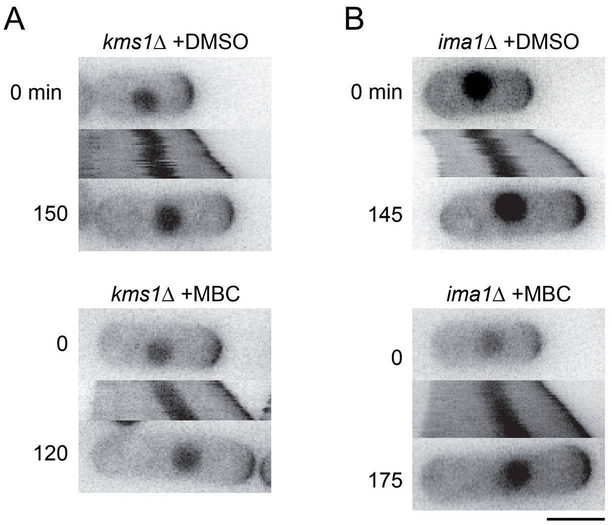

Figure S5. MINM occurs in *kms1*Δ and *ima1*Δ cells.
(A, B) Timepoints and kymographs of nuclear movement from DMSO- and MBC-treated *kms1*Δ (A) and *ima1*Δ (B) cells. Timepoints (min) correspond to beginning and end of kymographs. MINM was observed in 30 out of 30 MBC-treated *kms1*Δ cells and in 30 out of 30 *ima1*Δ cells. Bar, 5 µm.

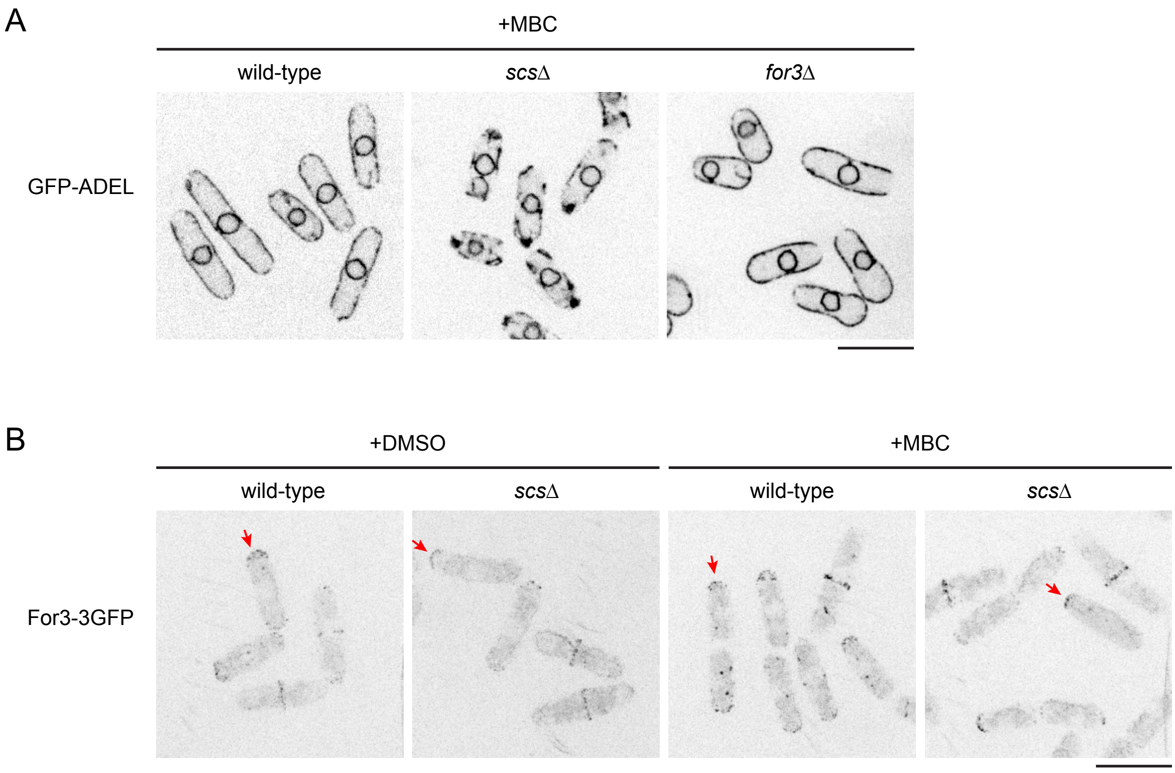

Figure S6. ER distribution in *for3*Δ cells and For3-3GFP localization in *scs*Δ cells.
(A) Distribution of endoplasmid reticulum (ER)/nuclear envelope (NE) reporter GFP-ADEL (Zhang et al., 2012) in wild-type, *scs*Δ, and *for3*Δ cells after 45 min MBC treatment. Images are single Z-sections from middle of cells. Distribution of ER/NE is similar to that previously described by Zhang et al. in the absence of MBC treatment (Zhang et al., 2012); in *for3*Δ cells, ER does not always reach as far to the cell tip as ER in wild-type cells, and in *scs*Δ cells, ER is detached from the plasma membrane. Experiments in wild-type cells indicate that GFP-ADEL localization is identical whether the *pBip1:GFP-ADEL* reporter gene is integrated at the l*eu1* locus (as shown here) or at the u*ra4* locus. (**B)** For3-3GFP localization in wild-type and s*cs*Δ cells after 45 min DMSO or MBC treatment. In all cases, For3-3GFP is localized primarily to cell tips during interphase (arrows) and to the cell middle during cell division. After MBC treatment, an increased number of For3-3GFP puncta away from cell tips is observed in wild-type cells compared to s*cs*Δ cells, although For3-3GFP is still retained at cell tips in wild-type cells. It is possible that this difference is somehow related to the accumulation of ER seen at cell tips in s*cs*Δ cells (A), but whether it has any physiological significance is unknown. Currently we do not envision that is has any direct relevance to MINM. Bar, 10 μm.

Journal of Cell Science • Supplementary information

Table S1. Yeast strains used in this study, listed by figure

Note: Strains used in more than one figure are listed more than once.

Fig. 1

Strain number	Genotype	Source
KS7305	*h- pAdh13:CRIB-3mCitrine:LEU2 ade6-M210 ura4-D18 leu1-32*	Lab stock (Mutavchiev et al. 2016)
KS5372	*h+ uch2-mCherry:ura4+ hphMX6:nmt81-GFPatb2 ura4-D18 leu1-32 ade6*	Lab stock

Fig. 2

Strain number	Genotype	Source
KS7340	*h- pAdh13:CRIB-3mCitrine:LEU2 for3Δ:KanMX6 ade6-M210 ura4-D18 leu1-32*	This study
KS7465	*h+ for3-3GFP-ura4+ pAdh13:CRIB-3mCitrine:LEU2 ade6-M216 leu1-32 ura4-D18*	This study
KS7466	*h- for3-I930A-3GFP-ura4+ pAdh13:CRIB-3mCitrine:LEU2 ade6-M210 ura4-D18 leu1-32*	This study

Fig. 3

Strain number	Genotype	Source
KS7305	*h- pAdh13:CRIB-3mCitrine:LEU2 ade6-M210 ura4-D18 leu1-32*	Lab stock (Mutavchiev et al. 2016)

Fig. 4

Strain number	Genotype	Source
KS7363	*h- myo51Δ::ura4+ pAdh13:CRIB-3mCitrine:LEU2 ade6-M210 ura4-D18 leu1-32*	This study
KS7366	*h+ pAdh13:CRIB-3mCitrine:LEU2 myo52Δ::ura4+ ade6-M216 leu1-32 ura4-D18*	This study
KS7514	*h+ pAdh13:CRIB-3mCitrine:LEU2 myo52Δ::ura4+ myo51Δ::KanMX6 ade6-M216 leu1-32 ura4-D18*	This study

Fig. 5

Strain number	Genotype	Source
KS7305	*h- pAdh13:CRIB-3mCitrine:LEU2 ade6-M210 ura4-D18 leu1-32*	Lab stock (Mutavchiev et al. 2016)
KS7340	*h- pAdh13:CRIB-3mCitrine:LEU2 for3Δ:KanMX6 ade6-M210 ura4-D18 leu1-32*	This study
KS9108	*h? scs2Δ::ura4+ scs22Δ::ura4+ pAdh13:CRIB-3mCitrine:LEU2 ade6? ura4-D18 leu1-32*	This study

Fig. 6

Strain number	Genotype	Source
KS7305	*h- pAdh13:CRIB-3mCitrine:LEU2 ade6-M210 ura4-D18 leu1-32*	Lab stock (Mutavchiev et al. 2016)
KS7340	*h- pAdh13:CRIB-3mCitrine:LEU2 for3Δ:KanMX6 ade6-M210 ura4-D18 leu1-32*	This study
KS9108	*h? scs2Δ::ura4+ scs22Δ::ura4+ pAdh13:CRIB-3mCitrine:LEU2 ade6? ura4-D18 leu1-32*	This study

Fig. S1

Strain number	Genotype	Source
KS10485	*h- nmt81:GST-NLS-mCherry:Leu+ Pbgs4+:Cher-12A-bgs4+:leu1+ bgs4Δ::ura4+ ade6-216 leu1-32 ura4-D18*	This study

Fig. S2

Strain number	Genotype	Source
KS7566	*h+ pAdh13:CRIB-3mCitrine:LEU2 [pAct1-lifeact-mCherry-leu1+] ade6-M216 ura4-D18 le*	Lab stock (Mutavchiev et al. 2016)
KS8568	*h? for3Δ::KanMX6 pAdh13:CRIB-3mCitrine:LEU2 [pAct1-lifeact-mCherry-leu1+] ade6-M216 ura4-D18 leu1-32*	This study

Fig. S3

Strain number	Genotype	Source
KS7305	*h- pAdh13:CRIB-3mCitrine:LEU2 ade6-M210 ura4-D18 leu1-32*	Lab stock (Mutavchiev et al. 2016)
KS7340	*h- pAdh13:CRIB-3mCitrine:LEU2 for3Δ:KanMX6 ade6-M210 ura4-D18 leu1-32*	This study
KS9108	*h? scs2Δ::ura4+ scs22Δ::ura4+ pAdh13:CRIB-3mCitrine:LEU2 ade6? ura4-D18 leu1-32*	This study

Fig. S4

Strain number	Genotype	Source
KS6716	*natMX6:Z:ADH15:mCherry-Atb2 ade6 leu1-32 ura4-D18 h+*	Lab stock
KS8570	*for3Δ::KanMX6 natMX6:Z:ADH15:mCherry-Atb2 ade6 leu1-32 ura4-D18 h+*	This study

Fig. S5

Strain number	Genotype	Source
KS7620	*h- ima1Δ::KanMX6 pAdh13:CRIB-3mCitrine:LEU2 ade6-M210 leu1-32 ura4-D18*	This study
KS7621	*h- kms1Δ::KanMX6 pAdh13:CRIB-3mCitrine:LEU2 ade6-M210 leu1-32 ura4-D18*	This study

Fig. S6

Strain number	Genotype	Source
KS8982	*h+ pBip1-GFP-ADEL::leu1+ ura4-D18 ade6*	S. Oliferenko lab strain SO4808
KS8983	*h- pBip1-GFP-ADEL::Ura4+ leu1-32 ade6*	S. Oliferenko lab strain SO5293
KS8988	*h- scs2Δ::ura4+ scs22Δ::ura4+ pBip1-GFP-ADEL::ura4+ leu1-32 ade6*	S. Oliferenko lab strain SO6917
KS8984	*h+ for3Δ::Kan+ pBip1-GFP-ADEL::leu1+ ura4-D18 ade6*	S. Oliferenko lab strain SO5422
KS7458	*h- for3-3GFP-ura4+ ade6-M216 leu1-32 ura4-D18*	S. Martin lab strain YSM423
KS10500	*h? for3-3GFP-ura4+ scs2Δ::ura4+ scs22Δ::ura4+ ade6-M21X leu1-32 ura4-D18*	This study.

Table S2. Oligonucleotides used in this study

Number	Sequence	Description	Source	Notes
OKS215	CCTCTTCCGACCATCAAGCATTTTATCC	KanMX6 screening primer	Sawin Lab	Forward primer
OKS216	GGTTGCATTCGATTCCTGTTTGTAATTG	KanMX6 screening primer	Sawin Lab	Forward primer
OKS231	CTAGGTAAATCGAAACATTTT	Rev screening primer for *ura4*	Sawin Lab	5' out primer
OKS1865	CTAGGTAAATCGAAACATTTT	Rev primer to screen *for3*Δ	Sawin Lab	
OKS2552	TTCCTTACCATTTATTCCTTAATCAGCTTCGTTAGTATCTTTTTTACAACCAAATTACCAGTTTGGTATGTTAATTCATACGGATCCCCGGGTTAATTAA	Fwd primer to generate *for3*Δ	This study	Deleted by inserting Kan
OKS2553	TCTTTCAGACAAATCGTCAATGTATGTAATGTACAGATATACTGTTCTAAAAATCCATCCTAGAAAGAACAATGGAGCAAGAATTCGAGCTCGTTTAAAC	Rev primer to generate *for3*Δ	This study	Deleted by inserting Kan
OKS2603	ACTGTTGCAAAGAGCAGCGGTGT	Fwd primer to screen *myo51*Δ	This study	Will detect band if *myo51* present
OKS2605	TCTTTCTAGGATTTTTATTTTG	Rev primer to screen *myo51*Δ	This study	Will detect band if *myo51* present
OKS2607	AAGATGAAAACGAAAACGAAACTG	Fwd primer to screen *myo52*Δ	This study	Will detect band if *myo52* present
OKS2609	GCCGTCTGGTTCGATTTATCAGCT	Rev primer to screen *myo52*Δ	This study	Will detect band if *myo52* present
OKS2624	TTATTCCTTCTTTTGATATAGTTTTCCTTTTATACCACAGAAGATATTTTATTTTCAAAAGAAAGTAATTAAAAATTGCTCGGATCCCCGGGTTAATTAA	Fwd primer to generate *myo51*Δ	This study	Deleted by inserting Kan
OKS2626	TACATAGCACATCGAAACTCAAGTTACCCGATTTATAACTTTATTCCTTCTTTTGATATAGTTTTCCTTTTATACCACAGCGGATCCCCGGGTTAATTAA	Rev primer to generate myo51Δ	This study	Deleted by inserting Kan
OKS2640	GCCTTTATATTAAAAATGATCTA	Rev primer to screen *myo52*Δ	This study	Used with OKS231 for *ura4+*
OKS2643	CTTTTTGAGGACTACAATGAA	Rev primer to screen *myo51*Δ	This study	Used with OKS216 for Kan
OKS2667	AGCACTGATTTTTTTTTTAGAAAAAAAAAATCTTTCGCTAGCATCTTCATTTTCGTCATTATTTATTAGTCGCCTAATTAGAATTCGAGCTCGTTTAAAC	Fwd primer to generate *kms1*Δ	This study	Deleted by inserting Kan
OKS2668	TATGTACAAAAAGTTGAAAAAGGGTAAAGCAATTTAAATCAGCACTGATTTTTTTTTTAGAAAAAAAAAATCTTTCGCTAGAATTCGAGCTCGTTTAAAC	Rev primer to generate *kms1*Δ	This study	Deleted by inserting Kan
OKS2669	ACTTGTTGTTTCCCTTTTTTTTTTTAAATTTGCACACACAGGATTCTATGAGAACTTTGCATTAAATGGTATAATGGGAACGGATCCCCGGGTTAATTAA	Fwd primer to generate *ima1*Δ	This study	Deleted by inserting Kan
OKS2670	GGATAAATTAATGAATGATTGGTTTGCAAAAGAATATATTCCATATTACTTTGCATCCACTTCTTTAAATAGTAACCAGAGAATTCGAGCTCGTTTAAAC	Rev primer to generate *ima1*Δ	This study	Deleted by inserting Kan
OKS2677	ATTCCATATTACTTTGCATCCA	Rev primer to screen *ima1*Δ	This study	Used with OKS215 for Kan
OKS2680	GGGGATAACCTCAACGATAATT	Rev primer to screen *kms1*Δ	This study	Used with OKS215 for Kan
OKS3607	AGTAATAAAATCAAGAGCCATTAA	Rev primer to screen *scs2*Δ	This study	Deletion has *ura4+* in opposite orientation, so this primer works with OKS231
OKS3609	CAATTTTCATCGTCATACTAAT	Rev primer to screen *scs22*Δ	This study	Deletion has *ura4+* in opposite orientation, so this primer works with OKS231

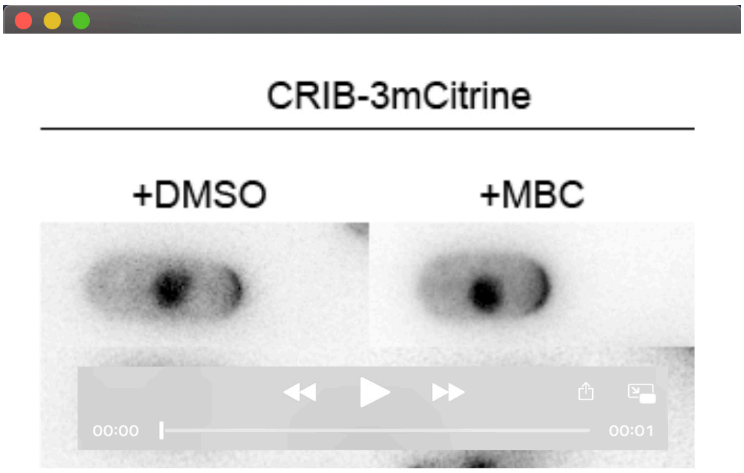

Movie 1. Microtubules are not required for nuclear movement in fission yeast. CRIB-3mCitrine in control (+DMSO) and MBC-treated wild-type cells. Cells correspond to those in Fig. 1D,E. Sequences vary in length. Time interval: 5 min. Total elapsed time of longest sequence: 230 min. Time compression at 15 frames per second playback: 4500x.

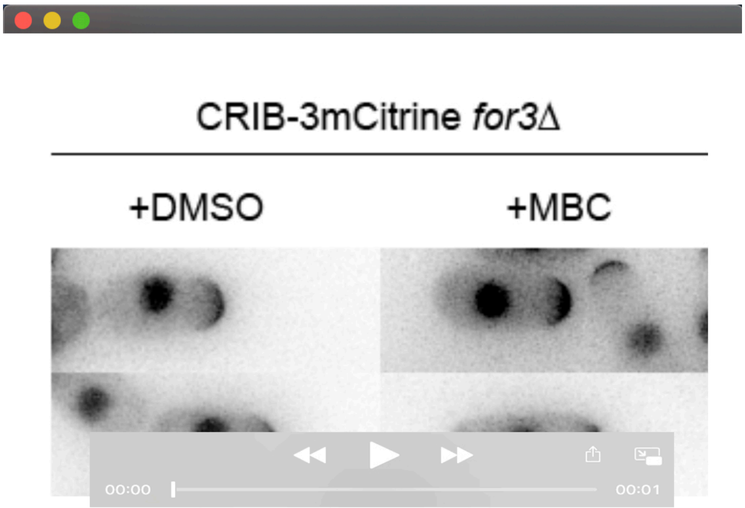

Movie 2. Microtubule-independent nuclear movement (MINM) requires formin For3. CRIB-3mCitrine in control (+DMSO) and MBC-treated *for3*Δ cells. Cells correspond to those in Fig. 2A,B. Sequences vary in length. Time interval:5 min. Total elapsed time of longest sequence: 210 min. Time compression at 15 frames per second playback: 4500x.

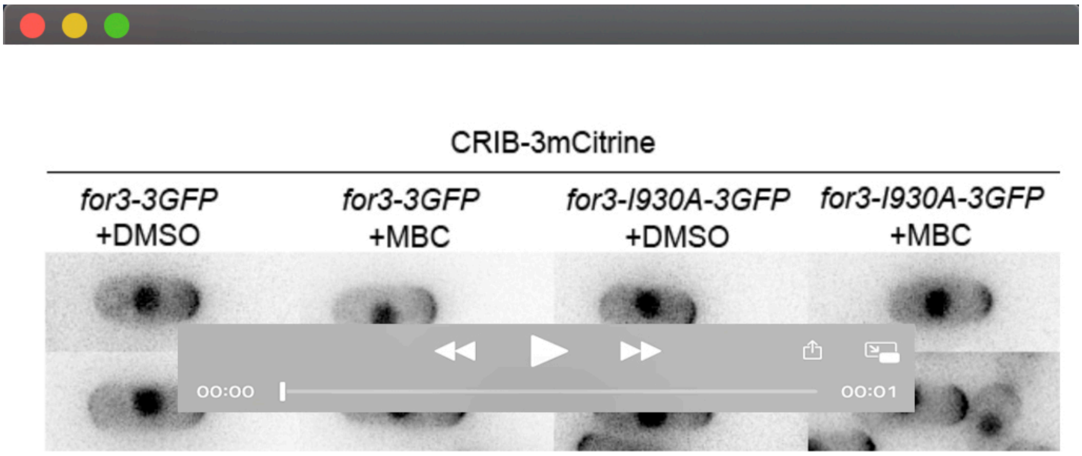

Movie 3. MINM requires For3's actin-nucleating activity.
CRIB-3mCitrine in control (+DMSO) and MBC-treated *for3-3GFP and for3-I930A-3GFP* cells. Cells correspond to those in Fig. 2C-F. Sequences vary in length. Time interval: 5 min. Total elapsed time of longest sequence: 225 min. Time compression at 15 frames per second playback: 4500x.

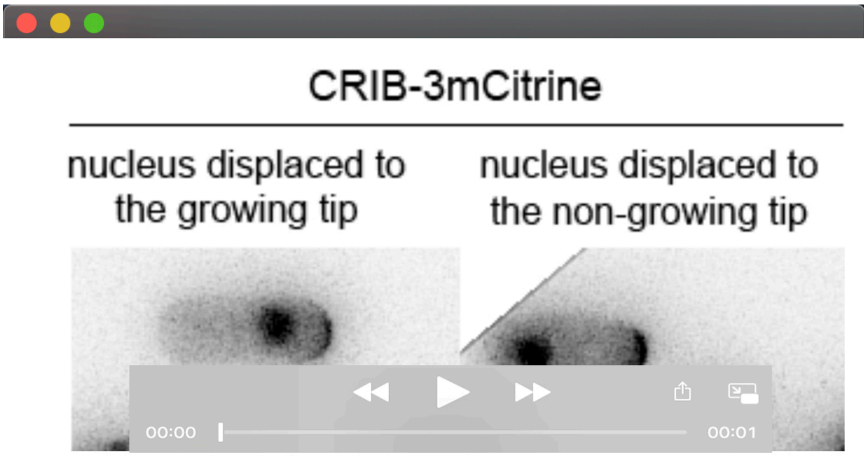

Movie 4. Velocity of MINM depends on distance of nucleus to growing cell tip.
CRIB-3mCitrine in wild-type cells treated with MBC, centrifuged to displace the nucleus, and then imaged in the continued presence of MBC. Cells correspond to those in top panels of Fig. 3B,C. Sequences vary in length. Time interval: 5 min. Total elapsed time of longest sequence: 170 min. Time compression at 15 frames per second playback: 4500x.

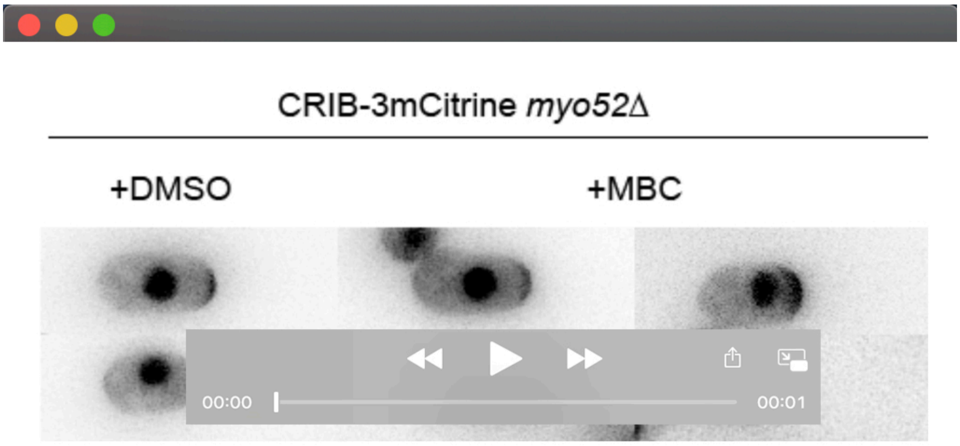

Movie 5. MINM persists in *myo52Δ* cells, although many cells show additional aberrant nuclear movements.
CRIB-3mCitrine in control (+DMSO) and MBC-treated *myo52Δ* cells. Middle panels show MINM in *myo52Δ* +MBC. Right-hand panels show aberrant nuclear movement in *myo52Δ* +MBC. Cells correspond to those in Fig. 4C,D. Sequences vary in length. Time interval: 5 min. Total elapsed time of longest sequence: 330 min. Time compression at 15 frames per second playback: 4500x.

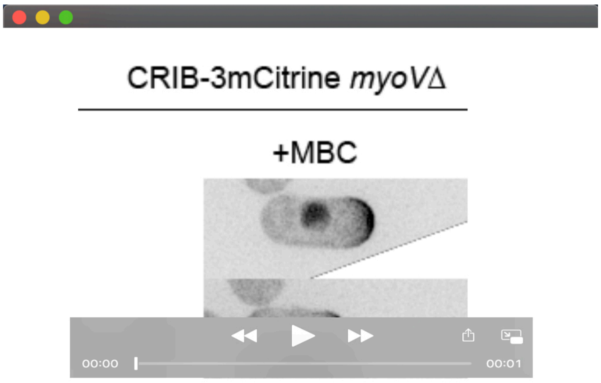

Movie 6. MINM persists in *myoVΔ* cells.
CRIB-3mCitrine in MBC-treated *myoVΔ* (i.e. double-mutant *myo51Δ myo52Δ*) cells. Cells correspond to those in Fig. 4E. Time interval: 5 min. Total elapsed time: 255 min. Time compression at 15 frames per second playback: 4500x.

Journal of Cell Science • Supplementary information

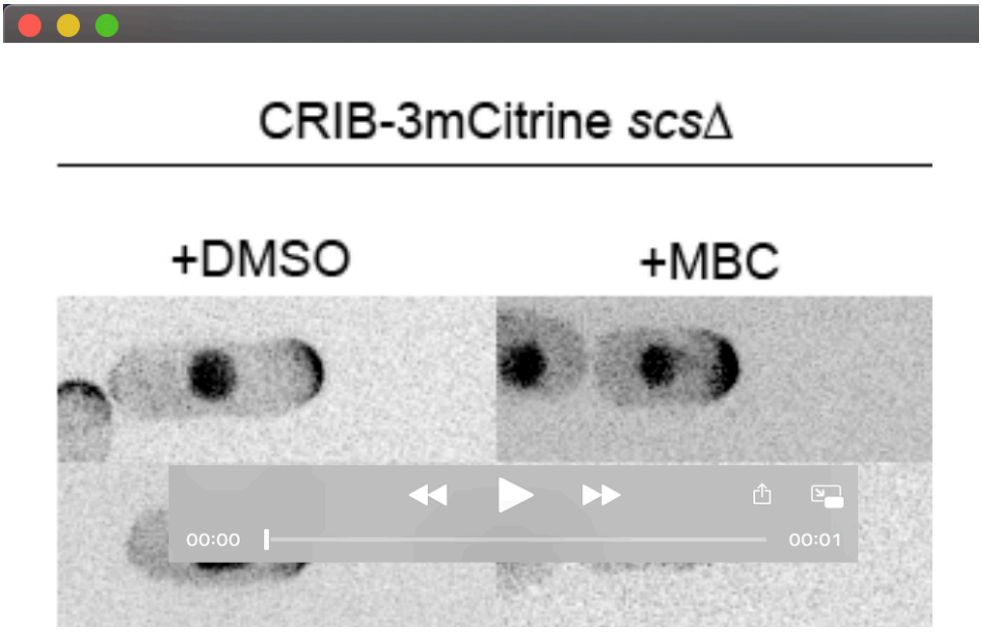

Movie 7. MINM requires VAPs Scs2 and Scs22.
CRIB-3mCitrine in control (+DMSO) and MBC-treated *scsΔ* (i.e. double-mutant *scs2Δ scs22Δ*) cells. Cells correspond to those in Fig. 5A,B. Sequences vary in length. Time interval: 5 min. Total elapsed time of longest sequence: 245 min. Time compression at 15 frames per second playback: 4500x.

Journal of Cell Science • Supplementary information
